# Treatment of Organics in Wastewater Using Electrogenerated Gaseous Oxidants

**DOI:** 10.1021/acs.iecr.3c03265

**Published:** 2024-04-09

**Authors:** Leticia Mirella da Silva, Ismael F. Mena, Cristina Saez, Artur J. Motheo, Manuel A. Rodrigo

**Affiliations:** † São Carlos Institute of Chemistry, University of São Paulo, P.O. Box 780, CEP 13560-970 São Carlos, SP, Brazil; ‡ Department of Chemical Engineering. Faculty of Chemical Sciences and Technologies, University of Castilla La Mancha, Campus Universitario s/n, 13071 Ciudad Real, Spain

## Abstract

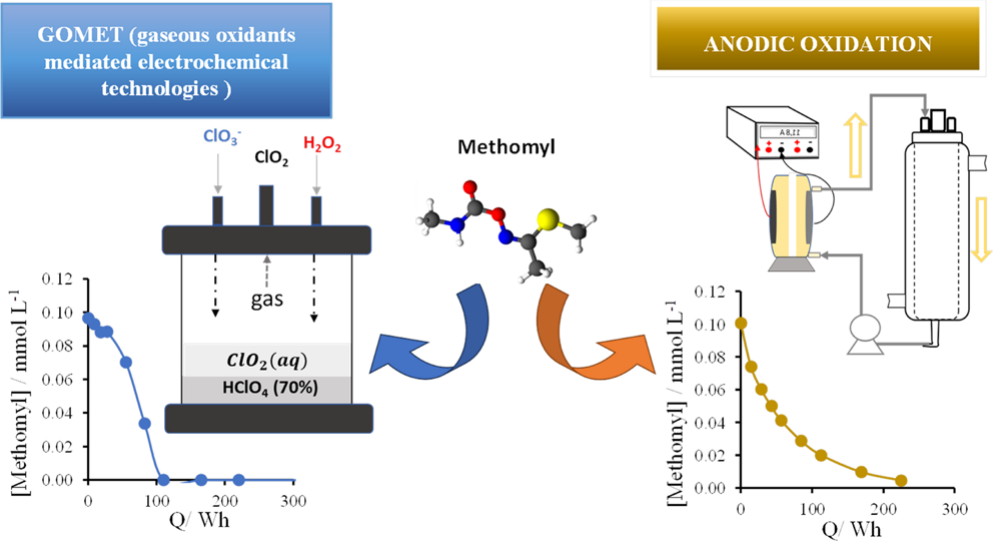

This work focuses on the comparison of the performance of direct electrochemical oxidation with indirect electrolysis mediated by gaseous oxidants in the treatment of diluted wastewater. To do this, energy consumptions of the electrolysis using mixed metal oxide (MMO) electrodes are compared with those required for the production and use of chlorine dioxide in the degradation of methomyl contained in aqueous solutions. Results demonstrate the feasibility of the mediated oxidation process and that this process is competitive with direct oxidation. The oxidants are produced under optimized conditions using the same anodic material applied for the direct degradation of organics, thus avoiding efficiency losses associated with mass transfer limitations in the degradation of dilute organic solutions. Thus, using the ClO_2_ gaseous oxidant, a concentration of 0.1 mM of methomyl from a solution containing 500 mL is completely removed with an energy consumption as low as 50 Wh. The application of the same energy to a direct electrolytic process for treating the same wastewater can only reach less than half of this removal. These findings may have a very important application in the use of electrochemical technology to achieve the remediation of persistent pollutants in wastewater, where their low concentrations typically make direct processes very inefficient.

## 1 Introduction

The removal of pollutants from wastewater has been a topic of wide interest for decades.^1−7^ Many technologies have been evaluated and, among them, electrochemical oxidation has been shown to be one of the most promising according to the scientific literature.^8−17^ In this context, once the viability of the electrochemical technology for treating organic compounds contained in wastewater was demonstrated at the turn of the century, other challenges were faced looking for improving efficiency and sustainability, including the development of new electrode materials,^18−24^ the synergistic effect and integration of electrolytic processes with other oxidation technologies,^25−30^ and the powering of electrochemical processes with green energies.^31^ More recently, technological advancement is focused on the development of more efficient electrochemical cells, with the final aim of increasing the technology readiness level (TRL) of these processes.^32^ In addition to this technological progress, fundamental studies that demonstrate the important role of hydroxyl radicals were considered extremely important to understand oxidation mechanisms and optimize their performance. The key role of these radicals has contributed to classify electrochemical oxidation as an advanced oxidation process (AOP).^33,34^

One of the most important facts that must be evaluated regarding the application of the electrochemical technology is the concentration of the organic pollutants that should be removed from wastewater.^35^ Typically, this technology is very efficient when treating wastewater with concentrations within the range of 1000–20,000 mg L^–1^ COD where, using diamond electrodes and tailored cells, Coulombic efficiencies near 100% can be reached.^36^ However, below this range, the efficiency decreases almost linearly with the concentration that needs to be removed. Thus, a typical efficiency when degrading wastewater with a COD content of 100 mg L^–1^ is around 5–10% and, in treating waste with a COD of 10 mg L^–1^, it is difficult to reach efficiencies over 2–3%, unless the bare electrolytic process is combined with other processes (Fenton, ultraviolet (UV) irradiation, application of ultrasounds, etc.).^37^ This is explained because the efficiency of the direct electrochemical processes depends importantly on mass transport within the electrochemical cell and below the range 1000–2000 mg L^–1^, this transport becomes the limiting stage (bottleneck), and it is necessary to change the primary oxidation mechanisms from direct transfer of electrons to a mediated process, to achieve a more efficient treatment.

The combination of electrochemical technologies with other advanced oxidation processes (AOPs) is this objective. The first works,^38,39^ made at a very low TRL of 2–3, aimed to clarify the effect of the salts contained in wastewater on the degradation of different pollutants (in fact, many of them were not real wastewater, but solutions containing the pollutant and salts in nonrealistic concentrations). Many works demonstrate the feasibility of removing the organic species in most cases, but the resulting effluent was not suitable for use in any case due to the difficulties in the separation of the oxidants and the salts contained in the electrolyte, as cited by Sirés et al.^40^ Then, the target of the research shifted from the evaluation of the effect of salts to the clarification of the role of other species such as hydrogen peroxide that do not leave an unaffordable fingerprint in the treated waste. The main disadvantage observed was the significant increase in cost when several technologies are combined, possibly associated with the fact that those works were carried out at a low TRL.^32^

Recently, interest has arisen in the electrochemical production of oxidants off-site from the waste treatment process, looking for higher efficiencies in this production, in which the electrolyte is not the waste but a special solution made on purpose to produce the oxidant.

Once formed, this oxidant is applied to the waste and it is let to oxidize the organic pollutants chemically, either alone or activated by chemical, photolytic, or ultrasound mechanisms.^41−47^ The main problem associated with this application is that, along with the addition of the oxidant, other compounds contained in the electrolyte where the oxidant was manufactured, are added to the water.^48−50^ Among these oxidant solutions, those containing peroxo species like peroxocarbonates, peroxosulfates, peroxophosphates, and hydrogen peroxide are worth to be mentioned. Also, and although used in much lower extension, hypochlorite solutions are also included. Thus, the main limitation in their use is the composition of the electrolyte used to manufacture them, because of the difficulties in the separation of the oxidants and the salts contained in the electrolyte in a cheap and efficient way.^40^

This problem can be detached when the oxidant produced is a gas, like in the cases of chlorine, ozone^51,52^ and chlorine dioxide,^53,54^ because in those cases, the electrolyte used for the production is not added to the waste that is expected to be treated, but simply a gaseous stream containing the oxidant gas, because they can be easily stripped from the electrolyte solution by flowing air and this becomes a very important advantage. This means that the fingerprint of this technology is going to be less important than that obtained with other more aggressively mediated processes. This approach is very new, especially in the case of chlorine dioxide, which is an oxidant whose electrochemical production has been the target of research only very recently. Although there are several approaches to produce this important oxidant,^55−58^ the most interesting path seems to be the combination of the production of chlorate (which is a well-known electrochemical process in which the raw matter can be brine or a salty chloride solution) (eqs 1–3) with the electrochemical production of hydrogen peroxide (in which a great development is being reached recently with the development of more and more efficient cells) (eq 4).^48,59,60^ In the combined process, the oxidation state of chloride (−1) is raised to that of chlorate (+5) electrochemically, and then hydrogen peroxide decreases it down to +4 (chlorine dioxide) (eq 5).^60−61,63^
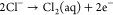
1
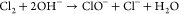
2
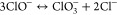
3
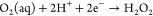
4
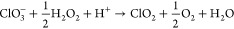
5

The ease in the change of oxidation state of chlorine species increases the difficulty of this process, which in addition needs highly acidic media. However, the good prospects of chlorine dioxide made it interesting to go further in the development of this electrochemical production.

Considering this background, the main aim of this paper is to make a preliminary comparison of the direct and mediated (using chlorine dioxide) degradation of pollutants at very low concentrations, especially in terms of energy demand. To do this, the degradation of a diluted solution containing methomyl is evaluated when the pollutant is degraded by direct electrolysis using mixed metal oxide (MMO) electrodes or when using for this degradation the oxidant chlorine dioxide, produced from chlorate and hydrogen peroxide, formed electrochemically with the same electrodes.

## 2 Materials and Methods

### 2.1 Chemicals

For the generation of oxidants, deionized water (Millipore Mili-Q system, resistivity 18.2 MΩ·cm at 25 °C, TOC:2 ppb) and the following analytical standard reagents were used: sodium chloride, sodium perchlorate, 70% perchloric acid, and 1.9–2.1% of titanium(IV) oxysulfate solution (CAS:13825–74–6/Sigma-Aldrich) as an indicator for H_2_O_2_ measurement and oxidants analysis, and the samples were acidified with 20% sulfuric acid for subsequent reaction with sodium thiosulfate. For the effluent preparation, 16.21 mg L^–1^ methomyl from its commercial compound was used, whose composition is 215 g L^–1^ methomyl, 420 g L^–1^ ethanol, and 324 g L^–1^ other ingredients.

### 2.2 Experimental Setup and Procedure

**Figure 1:**
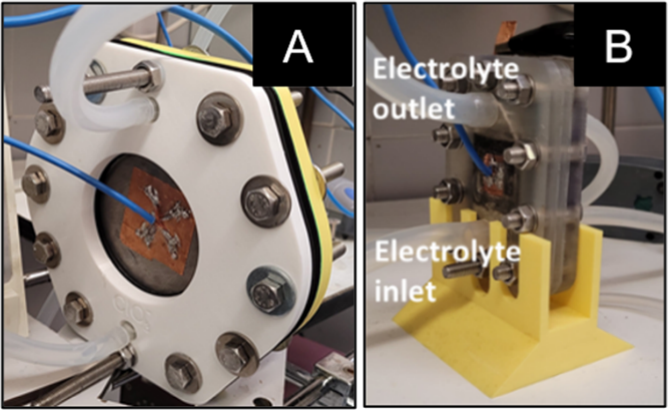
(A) Electrochemical flow reactor used for chlorate generation with a plate of titanium as cathode and DSA as an anode. (B) Gas diffusion cell used for hydrogen hydroxide generation with DSA as anode and painted carbon paper as cathode.

To generate chlorate from a sodium chloride solution, an electrochemical flow reactor (Figure 1A) manufactured with a three-dimensional (3D) printer was used, and it was equipped with an MMO (based on RuO_2_/Ti and supplied by Ti anode) anode and a titanium cathode, both measuring 78.5 cm^2^. The optimized conditions for production involved the use of a solution containing 5 g L^–1^ NaCl, applying 150 mA cm^–2^, at 15 °C, using a titanium plate as cathode. From this, it was possible to generate large amounts of ClO_3_^–^ continuously at different flow rates. The same MMO electrodes were used to produce chlorates and the direct degradation of methomyl.

To generate hydrogen peroxide (H_2_O_2_), an electrochemical gas diffusion cell (Figure 1B) printed on a 3D printer was used, using an MMO (based on RuO_2_/Ti and supplied by Ti anode) as the anode (10.9 cm^2^) and a painted carbon paper 10.9 cm^2^ as the cathode. The optimized condition used for methomyl treatment in this work was a solution of 14.05 g L^–1^ NaClO_4_ acidified to pH 3.5, applying 4.6 mA cm^–2^, with a continuous average flow of 100 mL h^–1^.

**Figure 2:**
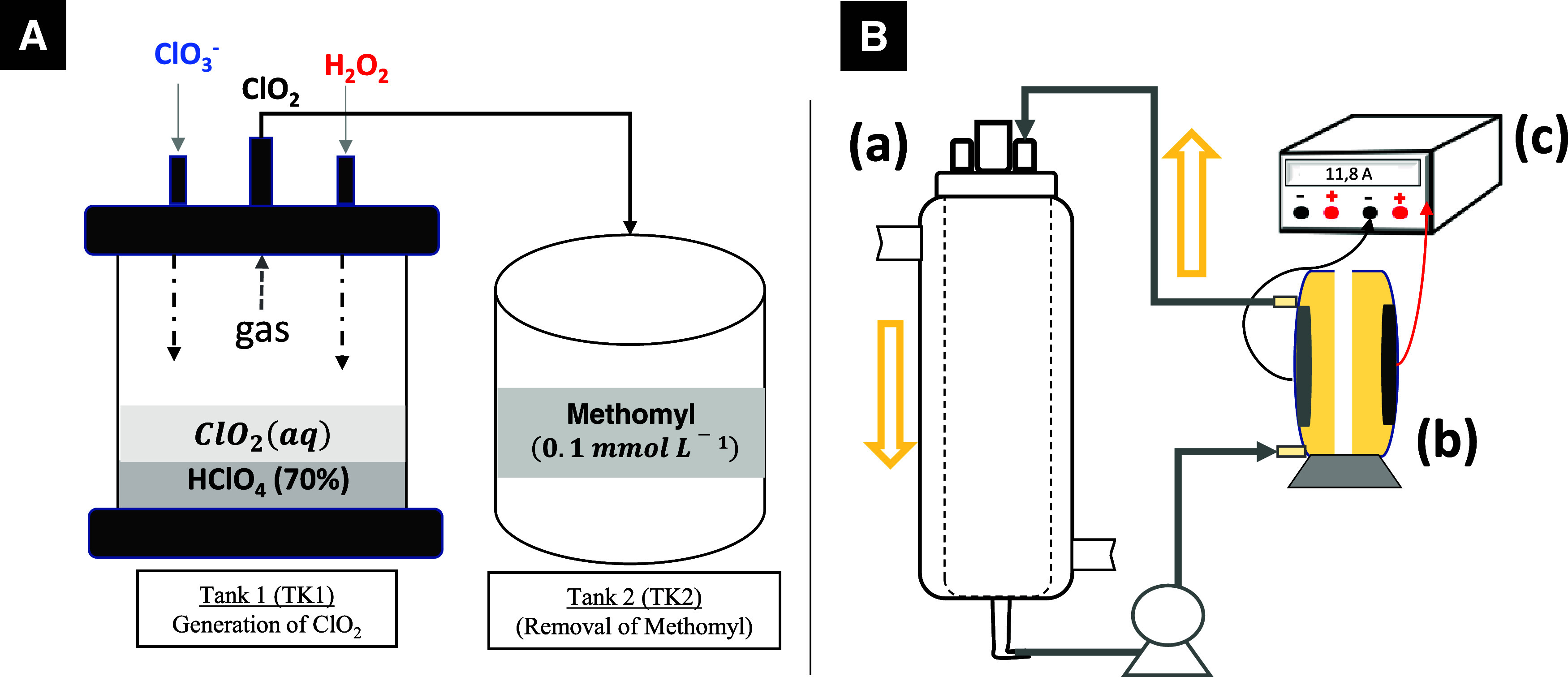
(A) Scheme of ClO_2_ production used for the treatment of methomyl in dissolution. (B) Electrochemical oxidation setup with MMO (Ti anode) anode: (a) recirculation tank with Na_2_SO_4_ (0.05 mol L^–1^), (b) electrochemical flow reactor, and (c) power supply. *j*_app_ = 79.5 mA cm^–2^, and initial volume of 500 mL.

As demonstrated in Figure 2A, the experiments of ClO_2_ generation were carried out in completely closed glass reactors of 1000 mL (Tank 1), adding different ratios of chlorate (4800 mg L^–1^) and hydrogen peroxide (46–70 mg L^–1^) simultaneously at different flow rates for ClO_2_ formation (eq 5). To maintain the strongly acidic pH, 100 mL of perchloric acid 70% were added in all cases as initial volume. Also, the H_2_O_2_ solution added was acid. Samples of the liquid and gas phases were collected to quantify the evolution of ClO_2_.

Two initial experiments were carried out with the system shown in Figure 2A. The first experiment (E1) used 2 L of KI (2 mol L^–1^) in TK2. The second experiment (E2) used 500 mL of a methomyl solution (0.1 × 10^–3^ mol L^–1^) in TK2. For ClO_2_ generation (TK1) was applied a continuous flow of 4.0 and 0.18 mmol h^–1^ for ClO_3_^–^ and H_2_O_2_, respectively, with an inlet airflow of 10 L h^–1^. Then, to evaluate the production of chlorine dioxide, the airflow and dosages of ClO_3_^–^ and H_2_O_2_ were varied as indicated in Table 1.

**Table 1:** ClO_2_ Generation Conditions, Using 100 mL of HClO_4_ (70%), and *V*_i_ = 500 mL

ID	airflow (mL h^–1^)	ClO_3_^–^ flow (mL h^–1^)	H_2_O_2_ flow (mL h^–1^)
(1)	50	62.5	92
(2)	30	62.5	92
(3)	10	62.5	92
(4)	30	67.9	187.5
(5)	30	180	64.3

The degradation of methomyl by electrochemical oxidation using MMO (RuO_2_/Ti) as anode and Ti anode as cathode was carried out using the system shown in Figure 2B, using a recirculation tank of 500 mL (a) with Na_2_SO_4_ (0.05 mol L^–1^), electrochemical flow reactor (b), and power supply (c) to apply 79.5 mA cm^–2^.

To calculate energy consumption associated with electrochemical processes, eq 6 was applied using intensity and voltage data obtained during the electrolysis tests. Energy data obtained are used to plot the main results of the methomyl removal.
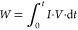
6

### 2.3 Analytical Techniques

The methomyl analysis was performed using a high-performance liquid chromatography (HPLC) method. For this purpose, an Elipse Plus C18 column from Agilent (PN: 959961–902) was used, with a mobile phase of acetonitrile:water (20:80, v/v), a flow of 0.3 mL min^–1^, 25 °C, injection volume of 20 μL, and λ_max_ = 233 nm. Chlorine dioxide (ClO_2_) in the liquid phase was monitored by spectrophotometry using a Spectroquant Prove 300 from Merck KGaA, D-64293 Darmstadt, with a wavelength absorbance of 360 nm. Previous experiments from our research group indicated the calibration curve of standard ClO_2._^60^ For the gaseous phase determinations, samples of 5 mL of gas were taken and bubbled into 10 mL of water for measuring the spectra or into a solution containing potassium iodide, producing its transformation into iodine. In this latter case, the iodine solution was titrated with sodium thiosulfate (0.001 N) to quantify oxidants.

## 3 Results and Discussion

### 3.1 Degradation of Methomyl by the Mediated Electrochemical Process

One of the main challenges in the development of new processes to face the destruction of organics contained in wastewater is to evaluate their performance in continuous operation mode, which is the operation mode in which full-scale treatment should be applied. This is of great significance when the product is a gas, and it is especially important in the case of chlorine dioxide because its production implies the integration of two electrochemical processes (production of chlorate and hydrogen peroxide) and one chemical step (reaction between both electrogenerated products). In previous works,^60,63^ it was shown how this process yields good results, especially with the use of tailored cells and suitable values of the key operational parameters. In this work, we start from the knowledge acquired in these previous works and design the tests to operate under conditions that were found to reach high efficiencies.

**Figure 3:**
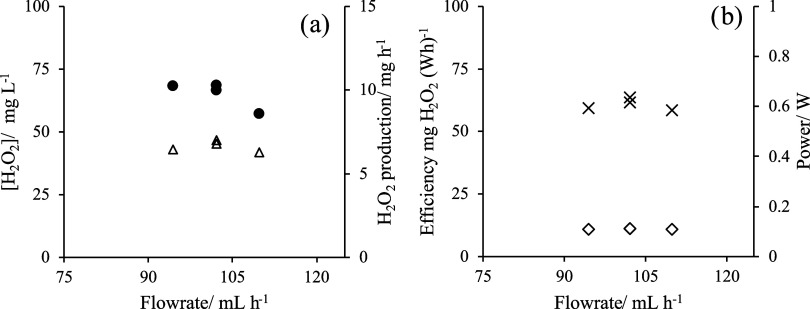
(a) H_2_O_2_ concentration (●) and H_2_O_2_ production (Δ) vs continuous flow rate (mL h^–1^). (b) Efficiency (×) and power (◊) calculated for H_2_O_2_ production.

It should be noted that Figure 3 shows the effect of the variability of the flow rate on the production of hydrogen peroxide using electrochemical technology within the nearness of the design conditions selected in this work, in which a cell with an electrode area of 9 cm^2^ was used (100 mL h^–1^) for the continuous production of H_2_O_2_. As seen, results are stable and with a tiny cell and extremely low energy consumption, it is possible to obtain a liquid stream of hydrogen peroxide with an average concentration over 60 mg L^–1^. Hence, as expected because of the dilution effect, the concentration of H_2_O_2_ decreases with the increase in the flow rate. This is not the case with the production that it is maintained in values higher than 6 mg h^–1^ regardless of the flow rate fed to the cell (with Faradaic efficiencies around 22.9%). In those conditions, power consumed is as low as 0.1 W, and thus, outstanding energy efficiencies are reached, in the nearness of 60 mg (Wh)^−1^.

**Figure 4:**
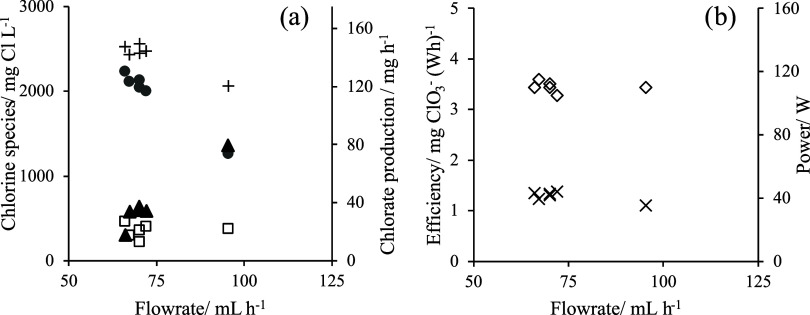
(a) Monitoring of chlorine species (mg Cl L^–1^), where the chlorine species are hypochlorite (□), chlorate (gray circle solid), and chloride (▲). Chlorate production (+, mg h^–1^) vs continuous flow rate (mL h^–1^). (b) Efficiency (×) and power (◊) calculated for chlorate production.

Figure 4 focuses on the continuous production of chlorate (in this case, at different flow rates in the nearness of 75 mL h^–1^, which was chosen as the design flow rate for an electrode surface area of 78.5 cm^2^), and it also shows the key parameters to understand this electrochemical process. A mixture of chlorine oxoanions is produced during the process, although in this case the effect of the flow rate is more relevant, and the higher the flow rate, the lower the concentration of chlorate in the resulting stream (same effect of that shown for hydrogen peroxide, although in this case is more clearly observed). Productions decrease with the flow rate, and values over 120 mg h^–1^ are obtained with power consumptions in the nearness of 110 W and energy efficiencies over 1.10 mg chlorate (Wh)^−1^, values much higher than those required by hydrogen peroxide production, making the energy required in that process negligible.

**Figure 5:**
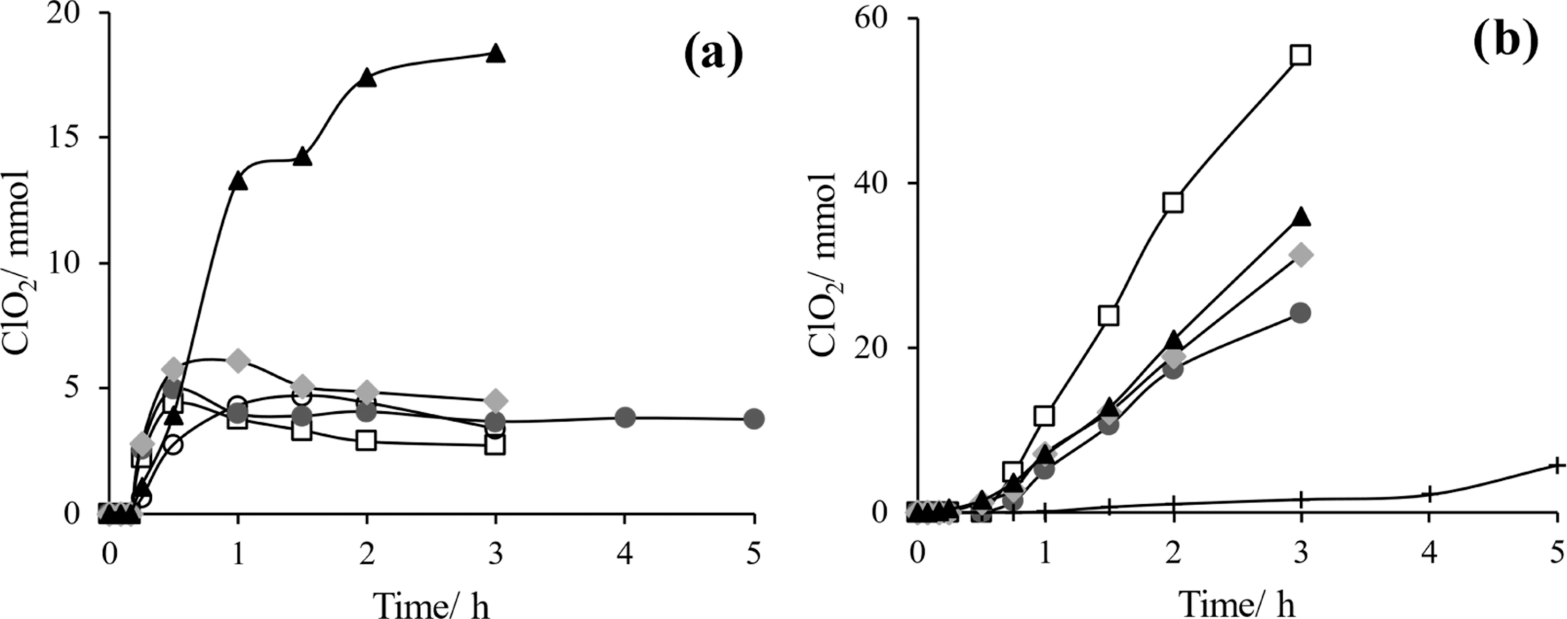
(a) Monitoring of the liquid chlorine dioxide produced (mmol) in the tank of reaction (tank 1) and (b) gas chlorine dioxide (mmol) diluted in the treatment tank with contaminated waste (tank 2). Conditions 1 (□), 2 (●), 3 (+), 4 (gray diamond solid), and 5 (▲) explained in Table 1.

Finally, the mixture of streams containing both species (in a strongly acidic perchlorate solution) yields chlorine dioxide both in the liquid and, especially, in a gas stream, as shown in Figure 5. Amounts of produced chloride dioxide largely depend on the dosing between electrochemically manufactured products and the airflow rate used to strip chlorine dioxide from the solution in which it is formed. The most significant parameter is the airflow rate, which informs about the rapid degradation of the chlorine dioxide in the liquid reaction media where it is produced, and of the necessity of exhausting it rapidly after being formed, especially if an efficient process is looked for. Hence, the most important parameter is not an electrochemical parameter but the flow rate of stripping air used that prevents decomposition of chlorine dioxide in the reaction tank where it is produced.

**Figure 6:**
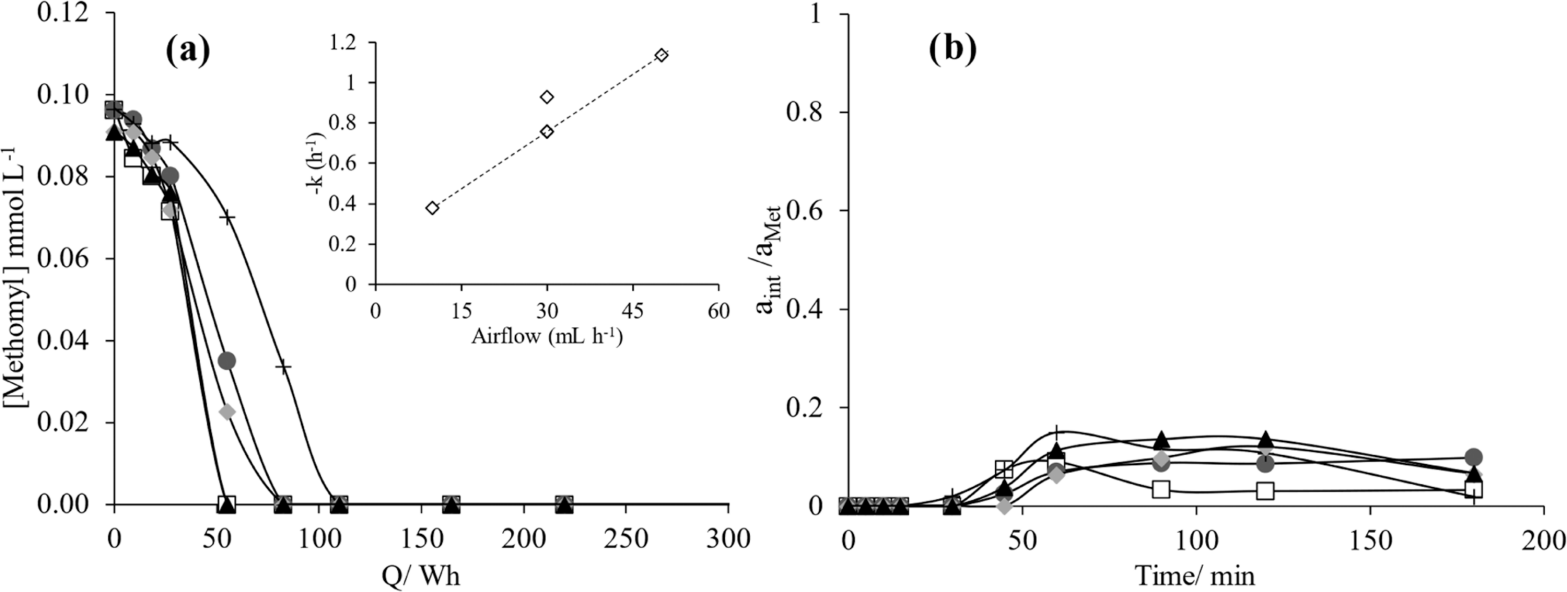
(a) Methomyl concentration decay (*C*_0_: 0.1 mmol L^–1^, V_i_: 500 mL) due to the ClO_2_ reaction under different conditions. *Inset:* The calculated constant rate vs airflow (mL h^–1^). (b) Chromatogram area of the sum of intermediates during the oxidation of methomyl using ClO_2_ produced. Conditions 1 (□), 2 (●), 3 (+), 4 (gray diamond solid), and 5 (▲) explained in Table 1. *a*_int_/*a*_Met_ is the area of intermediates divided by the area of Methomyl.

This gas containing ClO_2_ is flowed into synthetic wastewater polluted with methomyl. As seen in Figure 6a, the ClO_2_ stream rapidly produced a decay in the concentration of the pollutant, with a rate that is strongly dependent on the flow rate of gas flows (as shown in the onset). Chlorine dioxide is a very good oxidant of organic matter and, opposite to chlorine and hypochlorite, it does not produce chlorinated organic intermediates because the main process responsible for the formation of these species is prevented, as the chlorine addition reaction to the double bonds of organics does not happen with this oxidant as previously reported elsewhere.^64^ Results are plotted versus the energy applied for a later comparison that will be made with a direct electrolytic process (although as the production’s power is kept constant during the experiments, this trend is the same as that obtained concerning time). As seen in Figure 6a, energy consumption as low as 50 Wh allows for the total removal in the most efficient conditions, and in the worse conditions, the energy consumption is below 100 Wh. An intermediate is formed during the treatment (Figure 6b) and, as expected, it is rapidly depleted in the conditions in which more chlorine dioxide is added to the reactor. Hence, chlorine dioxide produced electrochemically can be used to degrade efficiently the methomyl contained in diluted solutions. TOC was measured for all samples, but no significant changes were observed and, in fact, the small changes detected could be associated with measurement errors rather than with real variations in this globalized parameter. This does not mean that no mineralization was obtained because it may be related to the complexity of the compounds contained in the commercial mixture used and hence their different impact on the TOC value (many different compounds for which the ratio TOC_measured_/TOC_theoretical_ is not 1, because of the inefficiencies in the oxidation made by the TOC analyzer).

### 3.2 Degradation of Methomyl by the Direct Electrochemical Process

**Figure 7:**
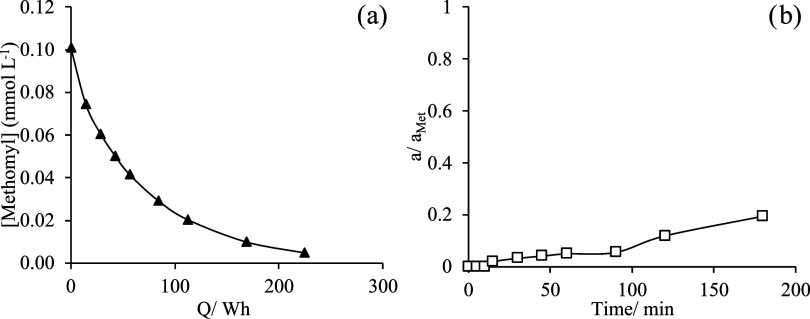
(a) Electrochemical removal of methomyl with the MMO (RuO_2_/Ti) anode (▲), using 0.1 mmol L^–1^ methomyl. (b) Chromatogram area of the sum of intermediates (□) formed during the electrochemical oxidation reaction of methomyl using the MMO (RuO_2_/Ti) anode. The supporting electrolyte was 0.05 mol L^–1^ Na_2_SO_4_, *j* = 79.5 mA cm^–2^, and *V*_i_ of 500 mL.

Using the same type of anodes that were used to produce chlorate (which was observed to be the bottleneck of the complete process), the electrolysis of the same methomyl solution, evaluated in this work as synthetic waste, was made. Typical operation conditions for this type of treatment were applied with a current density of 79.5 mA cm^–2^ and room temperature. The main results are summarized in Figure 7, where the decay in the concentration of methomyl and the total concentration of intermediates are shown in terms of energy consumption.

As seen, the decay is much less efficient in terms of energy, and more than 4 times more energy is required to complete the degradation of the methomyl solution as compared to the tests in which chlorine dioxide produced electrochemically is used as an oxidant (200 vs 50–100 Wh, respectively). However, it can be confirmed that anodic oxidation can also completely deplete methomyl from the liquid solution. Opposite to what was obtained when bubbling chlorine dioxide in the synthetic solution, the intermediates detected in those tests did not behave as intermediates here but as a final product, although it was produced in the same range of concentrations that reached in the mediated oxidation tests, pointing out again the lower efficiency of the direct electrochemical process.

**Figure 8:**
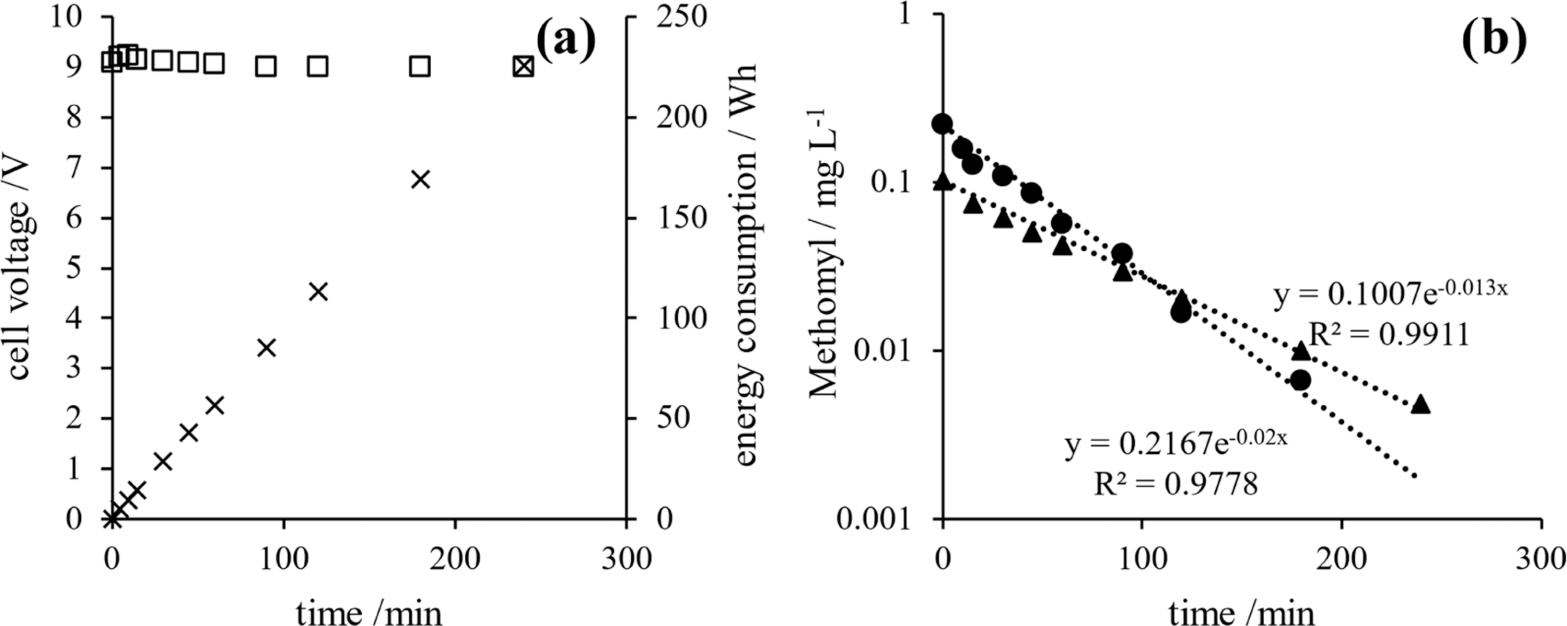
(a) Voltage (*V*, □) and energy consumption (Wh, ×) of the electrochemical oxidation process using MMO (RuO_2_/Ti) anode (*I* = 6.24 A). (b) Electrochemical removal of methomyl with MMO (RuO_2_/Ti) anode, using 0.2 and 0.1 mmol L^–1^ methomyl represented by (●) and (▲), respectively. The supporting electrolyte was 0.05 mol L^–1^ Na_2_SO_4_, *j* = 79.5 mA cm^–2^, and *V*_i_ = 500 mL.

Figure 8b compares the effect of the initial concentration in a semilogarithmic plot, where it can be seen that the process fits well to a first-order kinetic and that the first-order decay constant depends on the initial concentration as reported in the literature,^65,66^ pointing out the inefficiency in the application of anodic oxidation processes when treating diluted waste. A negligible effect was observed in the resulting cell voltage and energy applied to the system (Figure 8a), confirming that the efficiency of direct anodic oxidation processes increases linearly with the concentration. Thus, in this case, the energy efficiency of treating a solution with a concentration of 0.2 mg L^–1^ is 0.60 mg (kW h)^−1^, while in treating a solution with an initial concentration of 0.1 mg L^–1^ the efficiency decreases down to 0.268 mg (kW h)^−1^, both values much below the typically reported in studies in which concentrations are much higher.^67,68^ Also, Coulombic efficiencies considering an exchange of 26 electrons (nitrogen and sulfur as final products in the oxidation) lead to values of 1.81 and 0.78%, respectively, for the degradation of the solutions containing 0.2 and 0.1 mg L^–1^, values which can be explained in terms of the dilution, as the kinetics of the oxidation processes typically fits to a pseudo-first-order kinetics.

These results confirm the good prospects of the mediated electrochemical technology proposed for the treatment of dilute solutions and that they can become a real alternative to anodic oxidation, being less sensitive to the effect of dilution than the commonly evaluated direct electrochemical processes.

## 4 Conclusions

From this work, the following conclusions can be drawn:

- Methomyl can be removed electrochemically either in direct processes or from electrochemically produced chlorine dioxide. Direct removal fits well to a first-order kinetic model, but decay kinetic constants depend on the initial concentration of methomyl in the wastes.
- Chlorine dioxide can be produced from the mixture of chlorate and hydrogen peroxide streams produced electrochemically in continuous mode. The airflow rate used to strip chlorine dioxide was found to be a very important parameter in reaching high efficiencies.
- The efficiency of the anodic oxidation of methomyl solutions depends almost linearly on the initial concentration of pollutants in the treated wastewater. Chlorine dioxide gaseous oxidants mediated electrochemical technologies (GOMET) are more efficient than anodic oxidation for the remediation of diluted wastewater. It is possible to degrade methomyl with the same anode material and concentration in the synthetic wastewater with 4 times less energy. This indicates that the development of GOMETs is a topic worthy of further research.

## References

[ref1] López-Vizcaíno R.; Risco C.; Isidro J.; Rodrigo S.; Saez C.; Cañizares P.; Navarro V.; Rodrigo M. A. Scale-up of the Electrokinetic Fence Technology for the Removal of Pesticides. Part II: Does Size Matter for Removal of Herbicides?. Chemosphere 2017, 166, 549–555. 10.1016/j.chemosphere.2016.09.114. 27692679

[ref2] Gomes J. F.; Bednarczyk K.; Gmurek M.; Stelmachowski M.; Zaleska-Medynska A.; Bastos F. C.; Quinta-Ferreira M. E.; Costa R.; Quinta-Ferreira R. M.; Martins R. C. Noble Metal–TiO2 Supported Catalysts for the Catalytic Ozonation of Parabens Mixtures. Process Saf. Environ. Prot. 2017, 111, 148–159. 10.1016/j.psep.2017.07.001.

[ref3] Zhang W.; Sun W.; Zhang Y.; Yu D.; Piao W.; Wei H.; Liu X.; Sun C. Effect of Inorganic Salt on the Removal of Typical Pollutants in Wastewater by RuO2/TiO2 via Catalytic Wet Air Oxidation. Chemosphere 2023, 312, 137194 10.1016/j.chemosphere.2022.137194. 36372337

[ref4] Zhou M.; Tan Q.; Wang Q.; Jiao Y.; Oturan N.; Oturan M. A. Degradation of Organics in Reverse Osmosis Concentrate by Electro-Fenton Process. J. Hazard. Mater. 2012, 215–216, 287–293. 10.1016/j.jhazmat.2012.02.070. 22429623

[ref5] Abdessalem A. K.; Oturan M. A.; Oturan N.; Bellakhal N.; Dachraoui M. Treatment of an Aqueous Pesticides Mixture Solution by Direct and Indirect Electrochemical Advanced Oxidation Processes. Int. J. Environ. Anal. Chem. 2010, 90, 468–477. 10.1080/03067310902999132.

[ref6] Lin C.; Cheruiyot N. K.; Hoang H. G.; Le T. H.; Tran H. T.; Bui X. T. Benzophenone Biodegradation and Characterization of Malodorous Gas Emissions during Co-Composting of Food Waste with Sawdust and Mature Compost. Environ. Technol. Innov. 2021, 21, 101351 10.1016/j.eti.2020.101351.

[ref7] Feng L.; van Hullebusch E. D.; Rodrigo M. A.; Esposito G.; Oturan M. A. Removal of Residual Anti-Inflammatory and Analgesic Pharmaceuticals from Aqueous Systems by Electrochemical Advanced Oxidation Processes. A Review. Chem. Eng. J. 2013, 228, 944–964. 10.1016/j.cej.2013.05.061.

[ref8] Mansano A. S.; Moreira R. A.; Dornfeld H. C.; Freitas E. C.; Vieira E. M.; Sarmento H.; Rocha O.; Seleghim M. H. R. Effects of Diuron and Carbofuran and Their Mixtures on the Microalgae Raphidocelis Subcapitata. Ecotoxicol. Environ. Saf. 2017, 142, 312–321. 10.1016/j.ecoenv.2017.04.024. 28433596

[ref9] Sirés I.; Brillas E. Remediation of Water Pollution Caused by Pharmaceutical Residues Based on Electrochemical Separation and Degradation Technologies: A Review. Environ. Int. 2012, 40, 212–229. 10.1016/j.envint.2011.07.012. 21862133

[ref10] Miwa D. W.; Malpass G. R. P.; Machado S. A. S.; Motheo A. J. Electrochemical Degradation of Carbaryl on Oxide Electrodes. Water Res. 2006, 40, 3281–3289. 10.1016/j.watres.2006.06.033. 16914179

[ref11] Aquino J. M.; Rocha-Filho R. C.; Ruotolo L. A. M.; Bocchi N.; Biaggio S. R. Electrochemical Degradation of a Real Textile Wastewater Using β-PbO2 and DSA Anodes. Chem. Eng. J. 2014, 251, 138–145. 10.1016/j.cej.2014.04.032.

[ref12] Parra K. N.; Gul S.; Aquino J. M.; Miwa D. W.; Motheo A. J. Electrochemical Degradation of Tetracycline in Artificial Urine Medium. J. Solid State Electrochem. 2016, 20 (4), 1001–1009. 10.1007/s10008-015-2833-8.

[ref13] Labiadh L.; Fernandes A.; Ciríaco L.; Pacheco M. J.; Gadri A.; Ammar S.; Lopes A. Electrochemical Treatment of Concentrate from Reverse Osmosis of Sanitary Landfill Leachate. J. Environ. Manage. 2016, 181, 515–521. 10.1016/j.jenvman.2016.06.069. 27423100

[ref14] de Souza F.; Cristina S.; de Motheo A.; Rodrigo M. Electrochemical Removal of Dimethyl Phthalate with Diamond Anodes. J. Chem. Technol. Biotechnol. 2013, 89, 282–289. 10.1002/jctb.4118.

[ref15] Aquino J. M.; Pereira G. F.; Rocha-Filho R. C.; Bocchi N.; Biaggio S. R. Electrochemical Degradation of a Real Textile Effluent Using Boron-Doped Diamond or β-PbO2 as Anode. J. Hazard. Mater. 2011, 192 (3), 1275–1282. 10.1016/j.jhazmat.2011.06.039. 21742436

[ref16] Rocha I. M. V.; Silva K. N. O.; Silva D. R.; Martínez-Huitle C. A.; Santos E. V. Coupling Electrokinetic Remediation with Phytoremediation for Depolluting Soil with Petroleum and the Use of Electrochemical Technologies for Treating the Effluent Generated. Sep. Purif. Technol. 2019, 208, 194–200. 10.1016/j.seppur.2018.03.012.

[ref17] Sala M.; Gutiérrez-Bouzán M. C. Electrochemical Techniques in Textile Processes and Wastewater Treatment. Int. J. Photoenergy 2012, 2012, 629103 10.1155/2012/629103.

[ref18] Pupo M. M. S.; Da Costa L. S.; Figueiredo A. C.; Da Silva R. S.; Cunha F. G. C.; Eguiluz K. I. B.; Salazar-Banda G. R. Photoelectrocatalytic Degradation of Indanthrene Blue Dye Using Ti/Ru-Based Electrodes Prepared by a Modified Pechini Method. J. Braz. Chem. Soc. 2013, 24 (3), 459–472. 10.1590/S0103-50532013000300014.

[ref19] Berenguer R.; Sieben J. M.; Quijada C.; Morallón E. Pt- and Ru-Doped SnO2-Sb Anodes with High Stability in Alkaline Medium. ACS Appl. Mater. Interfaces 2014, 6 (24), 22778–22789. 10.1021/am506958k. 25453898

[ref20] Santos T. É. S.; Silva R. S.; Eguiluz K. I. B.; Salazar-Banda G. R. Development of Ti/(RuO2)0.8(MO2)0.2 (M = Ce, Sn or Ir) Anodes for Atrazine Electro-Oxidation. Influence of the Synthesis Method. Mater. Lett. 2015, 146, 4–8. 10.1016/j.matlet.2015.01.145.

[ref21] Wang Y.; Hu B.; Hu C.; Zhou X. Fabrication of a Novel Ti/SnO2–Sb–CeO2@TiO2–SnO2 Electrode and Photoelectrocatalytic Application in Wastewater Treatment. Mater. Sci. Semicond. Process. 2015, 40, 744–751. 10.1016/j.mssp.2015.06.020.

[ref22] da Silva L. M.; de Oliveira Santiago Santos G.; De Salles Pupo M. M.; Ivon K.; Eguiluz B.; Salazar-Banda G. R. Influence of Heating Rate on the Physical and Electrochemical Properties of Mixed Metal Oxides Anodes Synthesized by Thermal Decomposition Method Applying an Ionic Liquid. J. Electroanal. Chem. 2018, 813, 127–133. 10.1016/j.jelechem.2018.02.026.

[ref23] Malpass G. R. P.; de Jesus Motheo A. Recent Advances on the Use of Active Anodes in Environmental Electrochemistry. Curr. Opin. Electrochem. 2021, 27, 100689 10.1016/j.coelec.2021.100689.

[ref24] Vernasqui L. G.; dos Santos A. J.; Fortunato G. V.; Kronka M. S.; Barazorda-Ccahuana H. L.; Fajardo A. S.; Ferreira N. G.; Lanza M. R. V. Highly Porous Seeding-Free Boron-Doped Ultrananocrystalline Diamond Used as High-Performance Anode for Electrochemical Removal of Carbaryl from Water. Chemosphere 2022, 305, 135497 10.1016/j.chemosphere.2022.135497. 35764110

[ref25] Steter J. R.; Dionisio D.; Lanza M. R. V.; Motheo A. J. Electrochemical and Sonoelectrochemical Processes Applied to the Degradation of the Endocrine Disruptor Methyl Paraben. J. Appl. Eletrochem. 2014, 44, 1317–1325. 10.1007/s10800-014-0742-7.

[ref26] Domínguez J. R.; Muñoz-Peña M. J.; González T.; Palo P.; Cuerda-Correa E. M. Parabens Abatement from Surface Waters by Electrochemical Advanced Oxidation with Boron Doped Diamond Anodes. Environ. Sci. Pollut. Res. 2016, 23 (20), 20315–20330. 10.1007/s11356-016-7175-2. 27449015

[ref27] Montanaro D.; Lavecchia R.; Petrucci E.; Zuorro A. UV-Assisted Electrochemical Degradation of Coumarin on Boron-Doped Diamond Electrodes. Chem. Eng. J. 2017, 323, 512–519. 10.1016/j.cej.2017.04.129.

[ref28] Souza F. L.; Rocha R. S.; Ferreira N. G.; Rodrigo M. A.; Lanza M. R. V. Effects of Coupling Hybrid Processes on the Treatment of Wastewater Containing a Commercial Mixture of Diuron and Hexazinone Herbicides. Electrochim. Acta 2019, 328, 135013 10.1016/j.electacta.2019.135013.

[ref29] Dionisio D.; Motheo A. J.; Sáez C.; Rodrigo M. A. Effect of the Electrolyte on the Electrolysis and Photoelectrolysis of Synthetic Methyl Paraben Polluted Wastewater. Sep. Purif. Technol. 2019, 208, 201–207. 10.1016/j.seppur.2018.03.009.

[ref30] Li J.; Li Y.; Xiong Z.; Yao G.; Lai B. The Electrochemical Advanced Oxidation Processes Coupling of Oxidants for Organic Pollutants Degradation: A Mini-Review. Chin. Chem. Lett. 2019, 30 (12), 2139–2146. 10.1016/j.cclet.2019.04.057.

[ref31] Ganiyu S. O.; Martínez-Huitle C. A.; Rodrigo M. A. Renewable Energies Driven Electrochemical Wastewater/Soil Decontamination Technologies: A Critical Review of Fundamental Concepts and Applications. Appl. Catal., B 2020, 270, 118857 10.1016/j.apcatb.2020.118857.

[ref32] Lacasa E.; Cotillas S.; Saez C.; Lobato J.; Cañizares P.; Rodrigo M. A. Environmental Applications of Electrochemical Technology. What Is Needed to Enable Full-Scale Applications?. Curr. Opin. Electrochem. 2019, 16, 149–156. 10.1016/j.coelec.2019.07.002.

[ref33] Tufail A.; Price W. E.; Hai F. I. A Critical Review on Advanced Oxidation Processes for the Removal of Trace Organic Contaminants: A Voyage from Individual to Integrated Processes. Chemosphere 2020, 260, 127460 10.1016/j.chemosphere.2020.127460. 32673866

[ref34] Marselli B.; Garcia-Gomez J.; Michaud P.-A.; Rodrigo M. A.; Comninellis C. Electrogeneration of Hydroxyl Radicals on Boron-Doped Diamond Electrodes. J. Electrochem. Soc. 2003, 150 (3), D79 10.1149/1.1553790.

[ref35] Moreira F. C.; Boaventura R. A. R.; Brillas E.; Vilar V. J. P. Electrochemical Advanced Oxidation Processes: A Review on Their Application to Synthetic and Real Wastewaters. Appl. Catal., B 2017, 202, 217–261. 10.1016/j.apcatb.2016.08.037.

[ref36] Rodrigo M. A.; Cañizares P.; Sánchez-Carretero A.; Sáez C. Use of Conductive-Diamond Electrochemical Oxidation for Wastewater Treatment. Catal. Today 2010, 151 (1–2), 173–177. 10.1016/j.cattod.2010.01.058.

[ref37] Fryda M.; Matthée T.; Mulcahy S.; Höfer M.; Schäfer L.; Tröster I. Applications of DIACHEM Electrodes in Electrolytic Water Treatment. Electrochem. Soc. Interface 2003, 12, 40–44. 10.1149/2.F10031IF.

[ref38] Boudreau J.; Bejan D.; Bunce N. J. Competition between Electrochemical Advanced Oxidation and Electrochemical Hypochlorination of Acetaminophen at Boron-Doped Diamond and Ruthenium Dioxide Based Anodes. Can. J. Chem. 2010, 88 (5), 418–425. 10.1139/V10-017.

[ref39] Cañizares P.; Paz R.; Sáez C.; Rodrigo M. A. Electrochemical Oxidation of Alcohols and Carboxylic Acids with Diamond Anodes A Comparison with Other Advanced Oxidation Processes. Electrochim. Acta 2008, 53, 2144–2153. 10.1016/j.electacta.2007.09.022.

[ref40] Sirés I.; Brillas E.; Oturan M. A.; Rodrigo M. A.; Panizza M. Electrochemical Advanced Oxidation Processes: Today and Tomorrow. A Review. Environ. Sci. Pollut. Res. 2014, 21 (14), 8336–8367. 10.1007/s11356-014-2783-1. 24687788

[ref41] Steter J. R.; Dionísio D.; Rocha R. S.; Miwa D. W.; Lanza M. R.; Motheo A. J. Electrochemical Degradation of Methyl Paraben Using a Boron-Doped Diamond Anode. ECS Trans. 2012, 43 (1), 111–117. 10.1149/1.4704947.

[ref42] Lester Y.; Sharpless C. M.; Mamane H.; Linden K. G. Production of Photo-Oxidants by Dissolved Organic Matter during UV Water Treatment. Environ. Sci. Technol. 2013, 47 (20), 11726–11733. 10.1021/es402879x. 24011169

[ref43] Remucal C. K.; Manley D. Emerging Investigators Series: The Efficacy of Chlorine Photolysis as an Advanced Oxidation Process for Drinking Water Treatment. Environ. Sci. Water Res. Technol. 2016, 2 (4), 565–579. 10.1039/C6EW00029K.

[ref44] Wang J.; Wang S. Activation of Persulfate (PS) and Peroxymonosulfate (PMS) and Application for the Degradation of Emerging Contaminants. Chem. Eng. J. 2018, 334, 1502–1517. 10.1016/j.cej.2017.11.059.

[ref45] Lei Y. J.; Tian Y.; Fang C.; Zhan W.; Duan L. C.; Zhang J.; Zuo W.; Kong X. W. Insights into the Oxidation Kinetics and Mechanism of Diesel Hydrocarbons by Ultrasound Activated Persulfate in a Soil System. Chem. Eng. J. 2019, 378, 122253 10.1016/j.cej.2019.122253.

[ref46] Scialdone O.; Proietto F.; Galia A. Electrochemical Production and Use of Chlorinated Oxidants for the Treatment of Wastewater Contaminated by Organic Pollutants and Disinfection. Curr. Opin. Electrochem. 2021, 27, 100682 10.1016/j.coelec.2020.100682.

[ref47] Hand S.; Cusick R. D. Electrochemical Disinfection in Water and Wastewater Treatment: Identifying Impacts of Water Quality and Operating Conditions on Performance. Environ. Sci. Technol. 2021, 55 (6), 3470–3482. 10.1021/acs.est.0c06254. 33616403 PMC7970539

[ref48] Pinheiro V. S.; Paz E. C.; Aveiro L. R.; Parreira L. S.; Souza F. M.; Camargo P. H. C.; Santos M. C. Ceria High Aspect Ratio Nanostructures Supported on Carbon for Hydrogen Peroxide Electrogeneration. Electrochim. Acta 2018, 259, 865–872. 10.1016/j.electacta.2017.11.010.

[ref49] Llanos J.; Moraleda I.; Sáez C.; Rodrigo M. A.; Cañizares P. Electrochemical Production of Perchlorate as an Alternative for the Valorization of Brines. Chemosphere 2019, 220, 637–643. 10.1016/j.chemosphere.2018.12.153. 30599321

[ref50] Sánchez-Carretero A.; Sáez C.; Cañizares P.; Cotillas S.; Rodrigo M. A. Improvements in the Electrochemical Production of Ferrates with Conductive Diamond Anodes Using Goethite as Raw Material and Ultrasound. Ind. Eng. Chem. Res. 2011, 50 (11), 7073–7076. 10.1021/ie101438e.

[ref51] Bavasso I.; Montanaro D.; Di Palma L.; Petrucci E. Electrochemically Assisted Decomposition of Ozone for Degradation and Mineralization of Diuron. Electrochim. Acta 2020, 331, 135423 10.1016/j.electacta.2019.135423.

[ref52] Bavasso I.; Montanaro D.; Petrucci E. Ozone-Based Electrochemical Advanced Oxidation Processes. Curr. Opin. Electrochem. 2022, 34, 101017 10.1016/j.coelec.2022.101017.

[ref53] Ranieri E.; Świetlik J. DBPs Control in European Drinking Water Treatment Plants Using Chlorine Dioxide: Two Case Studies. J. Environ. Eng. Landscape Manage. 2010, 18 (2), 85–91. 10.3846/jeelm.2010.10.

[ref54] Terhalle J.; Kaiser P.; Jütte M.; Buss J.; Yasar S.; Marks R.; Uhlmann H.; Schmidt T. C.; Lutze H. V. Chlorine Dioxide - Pollutant Transformation and Formation of Hypochlorous Acid as a Secondary Oxidant. Environ. Sci. Technol. 2018, 52 (17), 9964–9971. 10.1021/acs.est.8b01099. 29966411

[ref55] Monteiro M. K. S.; Monteiro M. M. S.; de Melo Henrique A. M.; Llanos J.; Saez C.; Dos Santos E. V.; Rodrigo M. A. A Review on the Electrochemical Production of Chlorine Dioxide from Chlorates and Hydrogen Peroxide. Curr. Opin. Electrochem. 2021, 27, 100685 10.1016/j.coelec.2020.100685.

[ref56] Xu M. Y.; Lin Y. L.; Zhang T. Y.; Hu C. Y.; Tang Y. L.; Deng J.; Xu B. Chlorine Dioxide-Based Oxidation Processes for Water Purification:A Review. J. Hazard. Mater. 2022, 436, 129195 10.1016/j.jhazmat.2022.129195. 35739725

[ref57] Özdemir K. Chlorine and Chlorine Dioxide Oxidation of Natural Organic Matter in Water Treatment Plants. Environ. Prot. Eng. 2020, 46 (4), 87–97. 10.37190/epe200407.

[ref58] Schijven J.; Teunis P.; Suylen T.; Ketelaars H.; Hornstra L.; Rutjes S. QMRA of Adenovirus in Drinking Water at a Drinking Water Treatment Plant Using UV and Chlorine Dioxide Disinfection. Water Res. 2019, 158, 34–45. 10.1016/j.watres.2019.03.090. 31015141

[ref59] Cordeiro-Junior P. J. M.; Lobato Bajo J.; Lanza M. R. D. V.; Rodrigo Rodrigo M. A. Highly Efficient Electrochemical Production of Hydrogen Peroxide Using the GDE Technology. Ind. Eng. Chem. Res. 2022, 61 (30), 10660–10669. 10.1021/acs.iecr.2c01669. 35941851 PMC9354083

[ref60] Monteiro M. K. S.; Moratalla Á.; Sáez C.; Dos Santos E. V.; Rodrigo M. A. Production of Chlorine Dioxide Using Hydrogen Peroxide and Chlorates. Catalysts 2021, 11 (12), 1478 10.3390/catal11121478.

[ref61] Moratalla Á.; Monteiro M. K. S.; Sáez C.; Dos Santos E. V.; Rodrigo M. A. Full and Sustainable Electrochemical Production of Chlorine Dioxide. Catalysts 2022, 12 (3), 315 10.3390/catal12030315.

[ref62] Monteiro M. K. S.; Moratalla Á.; Sáez C.; Dos Santos E. V.; Rodrigo M. A. Electrochemical Production of Hydrogen Peroxide in Perchloric Acid Supporting Electrolytes for the Synthesis of Chlorine Dioxide. Ind. Eng. Chem. Res. 2022, 61 (9), 3263–3271. 10.1021/acs.iecr.1c04845. 35300272 PMC8919508

[ref63] Monteiro M. K. S.; Moratalla Á.; Sáez C.; Santos E. V. D.; Rodrigo M. A. Towards the Production of Chlorine Dioxide from Electrochemically In-Situ Produced Solutions of Chlorate. J. Chem. Technol. Biotechnol. 2022, 97 (8), 2024–2031. 10.1002/jctb.7073.

[ref64] Hwang E.; Cash J. N.; Zabik M. J. Chlorine and Chlorine Dioxide Treatment to Reduce or Remove EBDCs and ETU Residues in a Solution. J. Agric. Food Chem. 2002, 50 (16), 4734–4742. 10.1021/jf020307c. 12137506

[ref65] Grgur B. N.; Mijin D. Ž. A Kinetics Study of the Methomyl Electrochemical Degradation in the Chloride Containing Solutions. Appl. Catal., B 2014, 147, 429–438. 10.1016/j.apcatb.2013.09.028.

[ref66] Oturan N.; Zhou M.; Oturan M. A. Metomyl Degradation by Electro-Fenton and Electro-Fenton-like Processes: A Kinetics Study of the Effect of the Nature and Concentration of Some Transition Metal Ions as Catalyst. J. Phys. Chem. A 2010, 114 (39), 10605–10611. 10.1021/jp1062836. 20828190

[ref67] Tony M. A.; Mansour S. A. Microwave-Assisted Catalytic Oxidation of Methomyl Pesticide by Cu/ Cu 2 O/CuO Hybrid Nanoparticles as a Fenton-like Source. Int. J. Environ. Sci. Technol. 2020, 17 (3), 161–174. 10.1007/s13762-019-02436-x.

[ref68] Raut-Jadhav S.; Pinjari D. V.; Saini D. R.; Sonawane S. H.; Pandit A. B. Intensification of Degradation of Methomyl (Carbamate Group Pesticide) by Using the Combination of Ultrasonic Cavitation and Process Intensifying Additives. Ultrason. Sonochem. 2016, 31, 135–142. 10.1016/j.ultsonch.2015.12.015. 26964933

